# Burden of Chronic Hemodialysis on the Ability to Work: Time for Action

**DOI:** 10.1055/s-0044-1786869

**Published:** 2024-06-21

**Authors:** Fayez AlHejaili, Muhammad N. Hashmi, Abdulkareem Alsuwaida, Ghada A. Ankawi, Shahad A. ALMehaideb, Anas A. Alsuwaida, Mohammed T. AlZahrani, Ali E. Shehadah, Hatem A. AlNasser

**Affiliations:** 1 Department of Nephrology, King Saud Bin Abdulaziz University for Health Sciences, Riyadh, Kingdom of Saudi Arabia; 2 Department of Medicine, King Saud University Medical City, King Saud University, Riyadh, Kingdom of Saudi Arabia; 3 Department of Medicine, College of Medicine, King Abdulaziz University, Jeddah, Kingdom of Saudi Arabia

**Keywords:** employment, diabetes, chronic dialysis, chronic kidney disease, end-stage renal disease

## Abstract

**Background** Understanding the factors that contribute to unemployment will help in the design of creative resolutions to enable hemodialysis patients to return to a productive life.

**Methods** We examined employment among 625 patients aged 18 to 60 years who were on hemodialysis in 8 dialysis units.

**Results** Overall employment was low among patients on chronic hemodialysis at 49.7%. Unemployment was significantly higher in women than in men (86.6% vs 17.1%, *p* < 0.0001). The employment rate was 70.5% for those with no diabetes and hypertension, 29.5% for those with diabetes, and 25.9% for those with diabetes and hypertension. Furthermore, the results of the Cox regression showed that the variables of gender, level of education, capability of driving, and diabetes were related to employment of patients.

**Conclusions** The majority of patients on hemodialysis are unemployed or exit paid employment due to early retirement. Patients with diabetes and women are a vulnerable population with a higher unemployment rate.

## Introduction

End-stage renal disease is a worldwide burden in several countries. Three of four people with end-stage renal disease are of working age (20–64 years old), and the prevalence of chronic kidney disease (CKD) in this age group is expected to increase. 1 Unemployment is one of life's most traumatic experiences for patients with chronic illness. In addition to financial loss, it can cause significant detrimental effects on the mental, social, and emotional wellbeing of patients. Similarly, unemployment also has a significant negative impact on the economy of a country, causing a lower gross domestic product. There is growing demand in the labor market for a skilled workforce, and accommodating those with chronic diseases to fill that gap will have a positive financial impact on the overall wellbeing of patients and guarantee the sustainability of the social security system. In Europe, the rate of labor market participation of people over 55 years old is estimated to rise by 14.8% in 2060. 2 An international study conducted by the Harvard School of Public Health estimates that in the United States, there will be a $47-trillion loss of output due to chronic diseases and mental diseases. 3

This study investigated the association between employment status with comorbidities and sociodemographic parameters among patients with CKD on hemodialysis. Our goal was to understand working-age people who have an increased risk for unemployment and, thereby, suggest interventions that promote hiring and empowerment of patients on dialysis.

## Study Design

The study included data from 625 hemodialysis patients in Saudi Arabia from 8 hemodialysis units. Data were collected between March and November 2022. The study included hemodialysis patients 18 years of age or older. Patients for whom data were missing on age, sex, or employment status were excluded. Additionally, students were not included in the study.

We collected information on social factors from medical questionnaires completed by clinical team members and self-administered patient questionnaires. Employment was defined for patients < 60 years of age as follows: patients working part- or full-time were classified as employed. Patients older than 60 years were excluded from the employment analyses because retirement age is 60 years in Saudi Arabia. Early retirement is when employees are permitted to draw their pension before the retirement age of 60 years due to sickness, disability, or other medical conditions. Education level was categorized into two groups: high school graduate or less and university graduate or more.

The research protocols were approved by King Khaled University Medical City ethics committees (E-21–6484). The study was conducted in accordance with the Declaration of Helsinki, and all participants gave informed consent.

### Statistical Analysis

Patient characteristics, including demographics and comorbidities, were reported for the overall sample as well as by employment status and education level. Cox regression was used to model the association between employment, sex, and education. Patient characteristics associated with employment modeling unemployed versus employed as a binary outcome were examined. Those who were retired or older than 60 years were excluded from the analysis. Univariate and multivariate logistic regression was used to estimate the odds ratio (OR) of patients' employment status and to explore the risk factors of unemployment among patients with end-stage renal disease. Stepwise regression using backward selection method was also utilized to conduct variable selection. The final model only included the variables that contributed significantly to the model. All statistical analyses were performed using SAS version 9.4 (SAS Institute, Cary, North Carolina, United States).

## Results

The analysis included 625 patients, and Table 1 shows the overall patient characteristics of the participants in the study. The rates of retirement were 3.9%, 31.6%, and 34% among those aged 18 to 45, 46 to 60, and more than 60 years, respectively. Even when restricted to patients < 60 years, those who were employed tended to be much younger (mean age, 46.3 vs 59.1 years) than those who were unemployed.

**Table 1:** Demographic and clinical characteristics of the participants

Age, y	58.3 ± 16.0
Sex, female, %	46.9%
Marital status	
Married	457 (74.6%)
Single	105 (17.1%)
Divorced	35 (5.7%)
Widow	16 (2.6%)
Dialysis duration, y	5.1 ± 4.4
Education level	
High school education	371 (71.6%)
Higher education	147 (28.4%)
Difficulties in walking	
No difficulties	377 (61.0%)
Cannot walk	107 (17.3%)
Use cane	119 (19.3%)
Leg amputation	15 (2.4%)
Comorbid conditions	
Hypertension	554 (89.1%)
Diabetes mellitus	
Type 2	305 (50.6%)
Type 1	55 (9.0%)
Diabetic retinopathy	131 (21.2%)
Legally blind	15 (2.4%)
Smoker	75 (12.2%)
Ex-smoker	86 (13.9%)
Hyperlipidemia	244 (39.0%)
Cause of ESRD	
Diabetes mellitus	265 (42.4%)
Hypertension	146 (23.4%)
Glomerular disease	50 (8.0%)
Unknown	99 (15.8%)
Other	65 (10.4%)
Heart disease	
Coronary artery disease	153 (24.9%)
CABG	29 (4.7%)
Congestive heart failure	42 (6.8%)
Valvular heart disease	30 (4.9%)
Atrial fibrillation	45 (7.3%)
Peripheral vascular disease	74 (12.0%)
Cerebrovascular disease	49 (7.9%)
Amputation	25 (4.0%)
Seizure disorder	24 (3.9%)
Depression	28 (4.6%)
Hepatitis B or C virus	23 (3.7%)

Abbreviations: CABG, coronary artery bypass grafting; ESRD, end-stage renal disease.

Note: Mean ± standard deviation, or number and % shown.

Among 518 patients for whom education data were available, 28.4% were university graduates and 71.6% were high school graduates or less. Patients with a university degree or more had a lower mean age than those with a high school degree or less (mean, 43.2 vs 55.4 years, *p* < 0.0001). Patients who were unemployed were also much more likely to be female than male (86.6% vs 17.1%, respectively) ( Table 2 ). University graduates were 23.8% of women vs 36.4% of men ( *p* = 0.01).

**Table 2:** Patient characteristics stratified by employment status

	Employed	Unemployed
Sex		
Male	98 (23.3%)	55 (13.1%)
Female	22 (5.2%)	246 (58.4%)
Dialysis duration (y)	4.4	5.8
Age group		
18–45 y	61 (24.9%)	63 (25.7%)
46–60 y	46 (18.8%)	75 (30.6%)
Marital status		
Married	87 (21.1%)	196 (47.5%)
Unmarried	33 (8.0%)	97 (23.5%)
Diabetes	33 (8.2%)	147 (36.4%)
Smoking status		
Active	35 (8.4%)	14 (3.4%)
Former	17 (4.1%)	12 (2.9%)
Never smoker	65 (15.7%)	272 (65.5%)
Hypertension	107 (25.5%)	265 (63.1%)
Hypercholesterolemia	45 (10.8%)	98 (23.6%)
Coronary artery disease	22 (5.3%)	68 (16.4%)
Cerebrovascular disease	6 (1.4%)	21 (5%)
Peripheral vascular disease	10 (2.4%)	23 (5.6%)
Diabetic retinopathy	21 (5.0%)	52 (12.4%)
Depression	3 (0.7%)	15 (3.6%)

Number (%) are shown.

Patient characteristics associated with employment modeling unemployed versus employed as a binary outcome are shown in Table 2 . The comorbid conditions tended to have a more negative impact on the employment rate. Those with no diabetes and/or hypertension were more likely to be employed than those with both comorbidities. The employment rate was 70.5% for those with no diabetes and hypertension, 29.5% among those with diabetes, and 25.9% for those with diabetes and hypertension.

Among the comorbidities and patient characteristics, gender, educational level, diabetes, and ability to drive showed statistical significance ( *p* < 0.05). On the contrary, difficulty in walking, peripheral vascular disease, marital status, and hypertension exhibited no statistically significant difference ( *p* > 0.05), as shown in Table 3 . Of all tested variables in univariate analysis, sex had the strongest association by far with employment; the OR of not being employed was 0.07 (95% confidence interval [CI], 0.04–0.12), i.e., men had approximately 14 times higher odds of being employed than women. Education, but not age group, was also strongly associated with employment; the OR of not being employed compared with the reference group of university graduates was 0.3 (95% CI, 0.15–0.59) for high school education or less. The predictors of *p* < 0.05 in the univariate analysis ( Table 3 ) were taken as independent variables. The multifactor logistic regression analysis found that gender ( *p* = 0.002), diabetes (0.04), educational level, and ability to drive were factors affecting the employment of patients on dialysis. Adjusting for other variables, history of diabetes was associated with lower employment rate (OR, 1.70; 95% CI, 1.03–2.78). Similarly, gender was found significantly associated with unemployment (OR, 0.11; 95% CI, 0.03–0.41) ( Table 4 ).

**Table 3:** The results of the univariate Cox regression model

Patient characteristic	Odds ratio (95% CI)	*p* -Value
Gender (female vs male)	0.07 (0.04–0.12)	<0.001
Difficult in walking (no vs yes)	0.38 (0.14–1.05)	0.80
Peripheral vascular disease (no vs yes)	0.66 (0.42–1.03)	0.89
Marital status (married vs other)	2.07 (1.42–3.01)	0.05
Education (high school vs higher education)	0.30 (0.15–0.59)	0.03
History of diabetes (no vs yes)	1.38 (1.02–1.87)	0.03
Able to drive a car (no vs yes)	0.08 (0.05–0.11)	<0.001
Hypertension (no vs yes)	0.81 (0.49–1.35)	0.5

Abbreviation: CI, confidence interval.

Note: Odds ratio (95% CI) of unemployment; model restricted to patients < 60 years old.

**Table 4:** Evaluating the adjusted effect of covariates on employment status of participants (unemployed, employed) with significant predictors using multivariate Cox regression model

Patient characteristic	Odds ratio	95% confidence interval
Female vs male	0.11	0.03–0.41
High school education vs higher education	0.52	0.31–0.86
Diabetes (no vs yes)	1.70	1.03–2.78
Able to drive (no vs yes)	0.35	0.20–0.63

Note: Odds ratio (95% confidence interval) of unemployment; model restricted to patients < 60 years old. Adjusted model is simultaneously adjusted for sex, educational level, diabetes, and ability to drive.

## Discussion

This study aimed to evaluate unemployment in patients on chronic hemodialysis in relation to patient characteristics. Understanding the contributing factors can facilitate addressing the identified barriers, providing support and creating a suitable work environment. Our study showed that many patients on hemodialysis are unemployed. The results showed very significant sex disparities for hemodialysis patients. Those with higher education tended to be more likely to be employed. The results also showed that, after adjusting for different variables, diabetes affects the employment of patients on in-center hemodialysis. Unfortunately, many patients have several comorbidities that increase their likelihood of not being employed. Many young working patients end up with early retirement.

Advanced CKD places an enormous burden on patients and their families and the health care system. In addition to the financial impact, employment contributes to quality of life among patients with CKD. 4 5 All creative initiatives supporting employees with CKD to facilitate sustainable employment should be encouraged. The health and economic impact of CKD in developing countries will aggravate existing economic challenges. Patients' and governments' responses to mitigate job loss are tightly constrained. The strategic response to unemployment should be unprecedented in terms of resources mobilized, scope, and ambition. It should lead to a new creative model that is conducive to resilience and sustainability to maintain those with chronic medical conditions, including those on dialysis. The national strategies may include but are not limited to professional training, entrepreneurship training, on-the-job trainings, flexible working environments, and wage guarantee funds. The clinical and legislative team can collaborate to tackle issues that might prevent patients from losing their jobs.

The results of our study showed that the number of employed hemodialysis patients was higher in males than in females. Women's greater longevity means that they are likely to sustain the deleterious impact of unemployment. Data from a study conducted in Taiwan showed that men had a higher percentage of employment than women. 6

Our study showed that the majority of women with end-stage renal disease are unemployed. The average 2021 unemployment rate for females based on 180 countries was 10.2% and 21.6% for Saudi women. 7 This shows the huge impact of CKD on the employment rate as 58% of women on dialysis were unemployed. National active programs to increase the labor force participation of women specifically need to be developed and executed.

Diabetes is a chronic lifelong disease and can lead to many physical, mental, and social health of patients. The more comorbidities that the patient has, the more likely that he or she will lose his or her job. The employment rates for nondiabetic and hypertensive patients was 70.5%, while it was 29.5% for patients with diabetes only; for those who had diabetes and hypertension, the rate was 25.9%. In this study, despite adjusting for different predictors, the unemployment rate of diabetic patients was 1.729 times higher than that of the nondiabetic patients. Several studies showed that diabetes significantly decreases employment probabilities in general populations. 8 9 10 Our results highlight the detrimental employment impact of diabetes in individual with end-stage renal disease. A study by Nazarov et al showed that the best strategies for returning to work for those with chronic disease are multidisciplinary interventions involving various health and work professionals. 11 We now face a paradigm shift in the clinical outcomes of dialysis from evidence-based medicine outcomes to patient-reported outcomes. 12 Driving the attention of the clinical team to outcomes directed to employment will help everyone involved to be creative in adopting the optimal mode of renal replacement therapy. In particular, we are seeing an improvement in the survival of patients on dialysis. 13

The model of renal replacement may help enhance the employment of patients with CKD. Employment status and work functioning among kidney transplant recipients are better than those among nontransplanted patients. 14 Home dialysis will help patients to have flexible timing for their treatment and avoid absenteeism for their dialysis sessions. Home hemodialysis is the new upcoming and thriving solution for a better outcome. It gives patients flexibility and independence with the treatment schedule. A study in Japan displayed survival, physical, and work rates higher than conventional hemodialysis. 15 Several studies in different countries have shown that peritoneal dialysis patients have a superior quality of life than those on hemodialysis. 16 17 18

Our research describes the prevalence of CKD and comorbidities based on their employment status, and data were collected from active patient records and direct patient interviews to ensure the accuracy of the data. Participants included in the study were from eight different dialysis units to ensure the true reflection of the community. The causal effects of having CKD on employment status, or vice versa, cannot be distinguished because of the use of cross-sectional data and the bidirectional nature of health and employment.

## Conclusion

The employment rate can be affected by social, wellbeing, financial, and sex disparities. Improved quality of life can be achieved with education, maintenance of a healthy wellbeing, and being offered choices to aid in maintaining a productive life for patients on renal replacement therapy. Individuals on dialysis can and do serve as highly productive members of the workforce. Every effort must be made to persuade the employer to accommodate individual on dialysis to effectively perform the vast majority of jobs. Individuals on dialysis, specifically, if they are diabetic, may need certain accommodations on the job to perform their work responsibilities effectively and safely.
